# 79885 Self-Weighing in Adolescents with Obesity: Attitudes of Teens and their Parents.

**DOI:** 10.1017/cts.2021.491

**Published:** 2021-03-30

**Authors:** Carolyn Bramante

**Affiliations:** University of Minnesota

## Abstract

ABSTRACT IMPACT: Obesity is a quickly growing pandemic that negatively impacts health, and clinicians and clinics must employ all evidence-based tools (such as self-monitoring) to help patients control their weight. OBJECTIVES/GOALS: The objective of this study was to understand patient and parent perspectives on using daily self-weighing for adolescents with obesity who are seeking obesity treatment. The secondary objective was to understand perspectives on connecting smart-scales to the electronic medical record for messaging, feedback, and reminders between visits. METHODS/STUDY POPULATION: Thirty adolescents with obesity who were seeking obesity treatment at a tertiary pediatric weight management clinic were recruited into a single-arm feasibility study to create and assess a connection between blue-tooth scales and the electronic medical record. These adolescents and their parents were then approached about conducting exit interviews about their experience in the feasibility study -- their perspectives on connecting bluetooth scales connected to the electronic health record and using daily self-weighing at home to help them reach their healthy weight goals. The interviews were conducted by a trained interviewer who was not a PI or Co-I on the feasibility study, recorded, and transcribed. The interviews were organized on themes including technical challenges, mood, stress, clinic feedback. RESULTS/ANTICIPATED RESULTS: The main theme expressed by participants and parents was related to past experiences of their weight loss journey. Sub-themes included: technical challenges with apps, parental involvement in weighing, being told by clinicians to weigh, and intervention impact. Most parents desired more directions and help with setting up the app connection to the EHR. Most parents did not ask their child daily about their weight status as they did not want to cause stress. Some adolescents found it stressful when parents asked about daily weight status; others found it helpful or at least not stressful. Most participants had never been advised by their clinician to regularly self-weigh. Most found it helpful to monitor their weight regularly. Most asked for reminders from clinic to weight and for feedback on weight between visits. DISCUSSION/SIGNIFICANCE OF FINDINGS: Overall, adolescents with obesity reported self-weighing as being helpful and most wanted some, but not daily, involvement from parents. Most parents wanted additional technological support to create the scale set-up. Nearly all parents and adolescents wanted the weights to be connected to clinic, and for there to be feedback from clinic on weight.

